# Black pleural effusion caused by a pancreaticopleural fistula secondary to chronic alcoholic pancreatitis exacerbation accompanying COVID‐19

**DOI:** 10.1002/ccr3.7245

**Published:** 2023-04-25

**Authors:** Yusuke Watanabe, Yukihisa Takeda, Reimi Mizushima, Takashi Okuma, Kazutoshi Toriyama, Junichi Iwamoto, Hiroyuki Nakamura, Kazutetsu Aoshiba

**Affiliations:** ^1^ Department of Respiratory Medicine Tokyo Medical University Ibaraki Medical Center Ibaraki Japan; ^2^ Department of Infection Prevention and Control Tokyo Medical University Hospital Tokyo Japan; ^3^ Department of Respiratory Medicine Tokyo Medical University Hospital Tokyo Japan; ^4^ Department of Gastroenterology and Hepatology Tokyo Medical University Ibaraki Medical Center Ibaraki Japan

**Keywords:** black pleural effusion, pancreaticopleural fistula, pancreatitis

## Abstract

Pancreaticopleural fistula should be considered in alcohol abusers with pleural effusion, which can exhibit a black color.

## Abstract 2

We report a case of black pleural effusion secondary to a pancreaticopleural fistula associated with chronic alcoholic pancreatitis exacerbation accompanying coronavirus disease 2019. Black pleural effusion is a rare entity and requires prompt diagnostic and therapeutic management. Pancreaticopleural fistula should be considered in alcohol abusers with black pleural effusion.

## 1 INTRODUCTION

Black pleural effusion is a rare entity and requires prompt diagnostic and therapeutic management. We report a case of black pleural effusion secondary to a pancreaticopleural fistula associated with chronic alcoholic pancreatitis exacerbation accompanying coronavirus disease 2019 (COVID‐19).

## 2 CASE HISTORY

**FIGURE 1:**
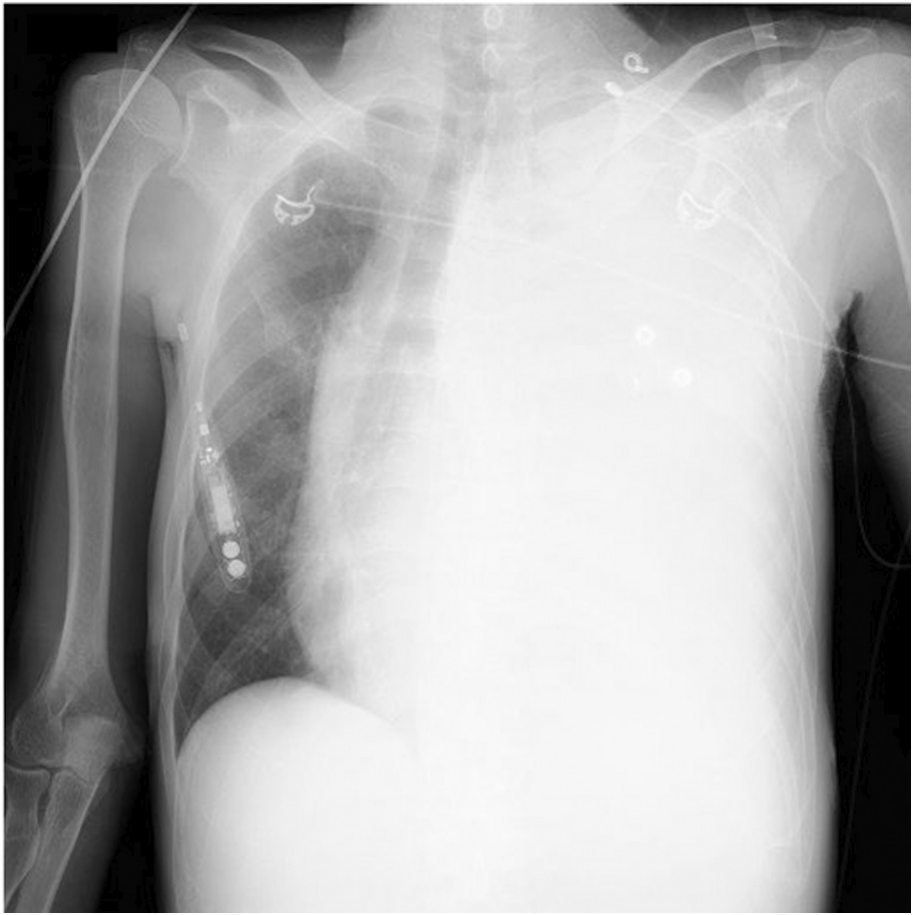
Chest X‐ray showed a left‐sided massive pleural effusion with a mediastinal shift to the right.

**FIGURE 2:**
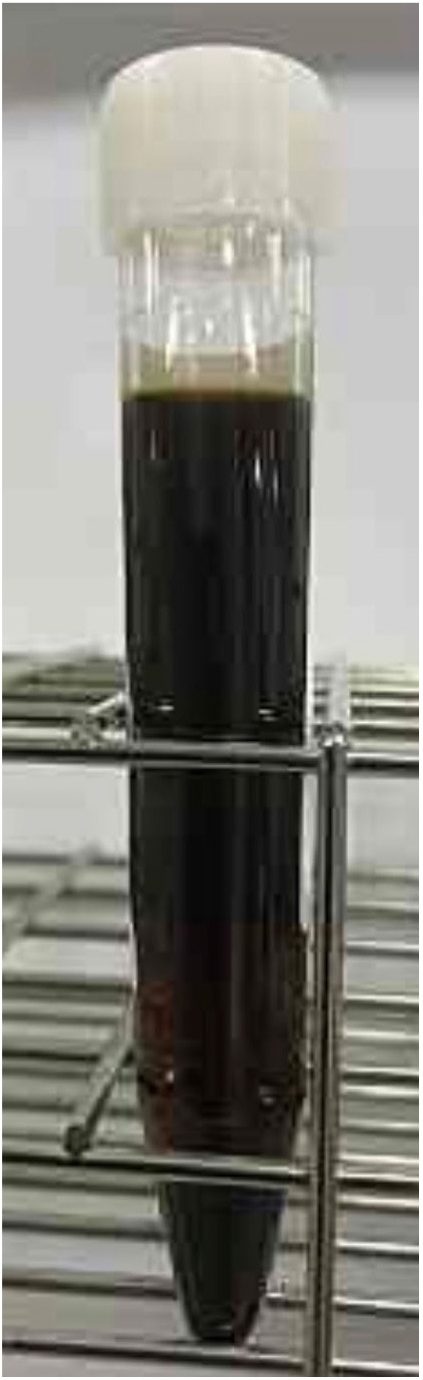
Black pleural fluid.

**FIGURE 3:**
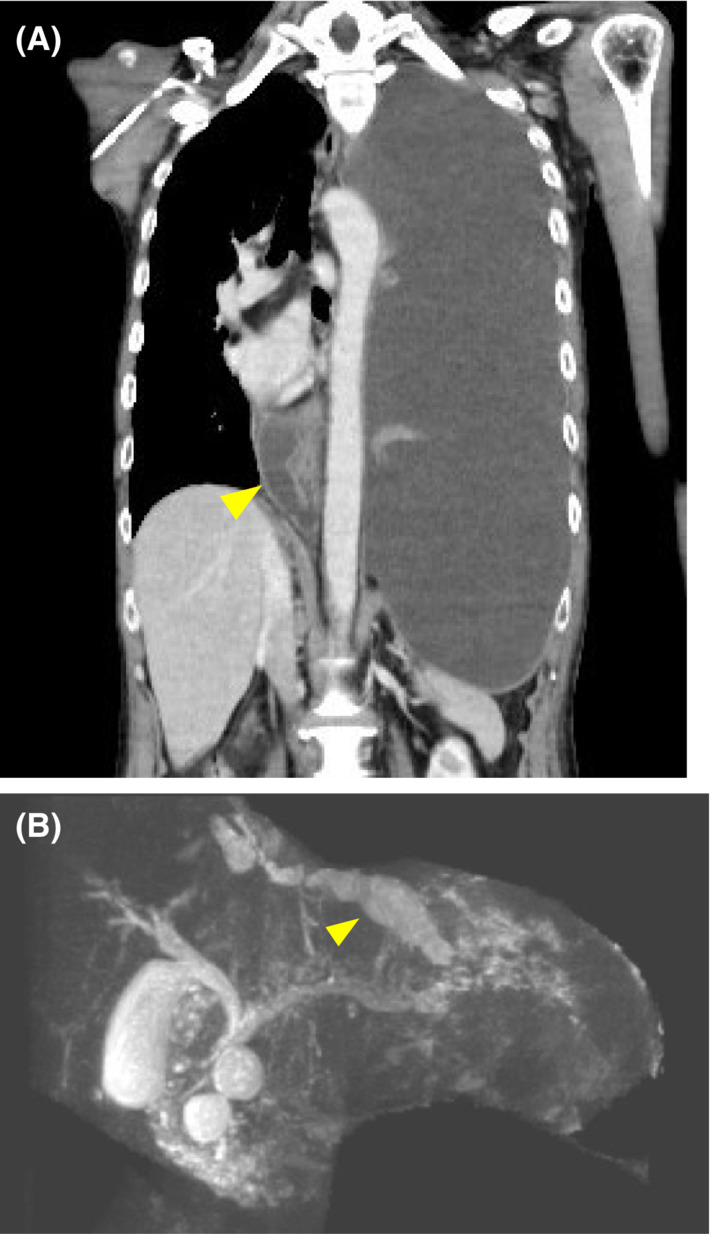
Contrast‐enhanced computed tomography (A) and plain magnetic resonance cholangiopancreatography (B) showed a pancreatic tail pseudocyst extending and connecting to the left pleural cavity through a mediastinal esophageal hiatal hernia (arrowheads).

A 57‐year‐old man with a known history of chronic alcoholic pancreatitis and severe acute respiratory syndrome coronavirus 2 (SARS‐CoV‐2) vaccination twice was admitted to our hospital with a 4‐day history of progressive exertional dyspnea. He was febrile, and his nasal swab for polymerase chain reaction test for SARS‐CoV‐2 was positive. He manifested with tachypnea (33 breaths per minute), tachycardia (140 beats per minute), and tenderness in the pericardial and epigastric area. Blood tests revealed an elevated amylase level (1319 U/L; normal: 44–132 U/L) with increased pancreatic type amylase (1292 U/L; normal: 18–57 IU/L). Chest X‐ray showed a massive left pleural effusion (Figure 1). Thoracocentesis revealed exudative black pleural effusion (Figure 2) with high levels of amylase (34,753 U/L), lipase (137,788 U/L), total bilirubin (2.4 mg/dL), and indirect bilirubin (2.2 mg/dL), suggestive of pancreatic enzyme‐induced hemorrhage and hemolysis. Thoracoabdominal contrast‐enhanced computed tomography (CT) (Figure 3A) and magnetic resonance cholangiopancreatography (Figure 3B) revealed a pancreatic pseudocyst, from which the encapsulated fluid extended to the left pleural cavity through a mediastinal esophageal hiatal hernia. There were no signs of COVID‐19 pneumonia.

## 3 DIAGNOSIS AND TREATMENT

Based on the above findings, a diagnosis of black pleural effusion due to a pancreaticopleural fistula secondary to alcoholic pancreatitis exacerbation accompanying COVID‐19 was established. The treatments included complete fasting, continuous pleural drainage through a chest tube, intravenous ulinastatin injection to inhibit pancreatic enzyme activity, and subcutaneous octreotide injection to inhibit pancreatic enzyme secretion. The patient had mild COVID‐19, and treatment nirmatrelvir/ritonavir showed improvement.

## 4 OUTCOME

Over the next 2 weeks, the amount of pleural discharge gradually decreased with improvement of symptoms. He was discharged on the 31st hospital day. Chest CT scan taken prior to discharge revealed pancreaticopleural fistula tract shrinkage and the absence of pleural fluid.

## 5 DISCUSSION

Black pleural effusion is a rare entity, which can be caused by fungal infection with *Aspergillus niger* or *Rhizopus oryzae*, metastatic melanoma, lung cancer, and pancreaticopleural fistula associated with pancreatic pseudocyst rupture.^1^ Pancreaticopleural fistula is observed in ~0.4% of patients with pancreatitis, most likely associated with alcohol abuse, and the pleural fluid contains a high amylase level. In our case, co‐infection with SARS‐CoV‐2 was incidentally found when pleural effusion was detected. We suspect that COVID‐19 may have exacerbated pre‐existing chronic alcoholic pancreatitis because pancreatic cells highly express angiotensin‐converting enzyme 2 receptors, which serve as the transmembrane proteins required for SARS‐CoV‐2 entry.^2^ COVID‐19 may have also induced pancreatic damage through systemic inflammatory responses, vasculitis, and thrombosis secondary to SARS‐CoV‐2 infection.^2^ During the COVID‐19 pandemic, a significant increase in alcohol consumption has been reported with an accompanying increase in the admission rates and mortality in patients with alcoholic pancreatitis.^3^

## 6 CONCLUSION

Pancreaticopleural fistula should be considered in alcohol abusers with pleural effusion, which manifest as a black discoloration.

## AUTHOR CONTRIBUTIONS

**Yusuke Watanabe:** Writing – original draft. **Yukihisa Takeda:** Writing – review and editing. **Reimi Mizushima:** Writing – review and editing. **Takashi Okuma:** Writing – review and editing. **Junichi Iwamoto:** Writing – review and editing. **Kazutetsu Aoshiba:** Conceptualization; supervision; writing – original draft.

## FUNDING INFORMATION

No sources of funding were used.

## CONFLICT OF INTEREST STATEMENT

The authors have no conflicts of interest to declare.

## CONSENT

Written consent from the patient was obtained for submission and publication of the case details and images from the patient. This case report meets the standards of Tokyo Medical University ethical committee.

## Data Availability

The data that support the findings of this study are available from the corresponding author upon reasonable request.
